# A Rhinolith Turning Out to Be an Intranasal Tooth

**DOI:** 10.7759/cureus.13616

**Published:** 2021-02-28

**Authors:** Hui Yan Ong, Jia Ji Ng, Hui Jun Ong, Shii, Joshua Wong, Shashi Gopalan

**Affiliations:** 1 Otolaryngology - Head and Neck Surgery, University of Malaysia Medical Centre, Kuala Lumpur, MYS; 2 Otorhinolaryngology, Universiti Kebangsaan Malaysia Medical Centre, Kuala Lumpur, MYS; 3 General Dentistry, Dental and Oral Health Division, Ministry of Health Malaysia, Kuala Lumpur, MYS; 4 Otorhinolaryngology, Hospital Tengku Ampuan Rahimah Klang, Klang, MYS

**Keywords:** rhinolith, foreign body, intranasal tooth, cleft palate

## Abstract

A tooth in the nasal cavity is an uncommon phenomenon. The exact mechanism is unclear, and patients may present with non-specific nasal symptoms. We encountered a 24-year-old patient with history of cleft palate repair, presenting to us with unilateral nasal discharge not improving with conventional medications. Rigid nasal endoscopy revealed a rhinolith-like foreign body at the floor of the left nasal cavity. Removal of the rhinolith was done under general anesthesia, and it turned out to be an intranasal tooth. Intranasal tooth is often misdiagnosed due to its non-specific symptoms. Detailed dental and oropharyngeal examination as well as imaging studies are essential in diagnosing an intranasal tooth. Early surgical removal is the mainstay of treatment in order to prevent further complications. Patients with unilateral nasal symptoms not responding to conventional treatment require proper ear, nose, and throat (ENT) evaluation to rule out other pathology.

## Introduction

The presence of tooth in the nasal cavity is a rare phenomenon, though it occurs more frequently in patients with associated cleft lip or cleft palate [1]. The exact mechanism of intranasal tooth is not well defined [2]. We encountered a 24-year-old female patient with a history of cleft palate repair, who presented to us with complaints of left nasal blockage and blood-stained nasal secretions for four months. Rigid nasal endoscopy revealed rhinolith on the floor of the left nasal cavity. Examination and removal of the rhinolith were done under general anesthesia. A tooth was found on the floor of the left nasal cavity with surrounding granulation tissue. The patient was discharged with no more nasal symptoms postoperatively.

## Case presentation

A 24-year-old female patient presented to us with complaints of left nasal obstruction, intermittent foul-smelling blood-stained nasal discharge, which was unprovoked for the past six months. There were no aggravating or relieving factors. She denied history of frequent rhinitis prior to that and had no associated symptoms such as frontal headache, facial pain, or facial numbness. She also had no ear symptoms. There were no history of trauma or foreign body insertion. She had a history of cleft palate defect during childhood for which palatal repair surgery was done. She was seen by her local general practitioners and was treated for rhinitis. Her symptoms were only alleviated slightly by steroidal nasal spray and antihistamines.

**Figure 1:**
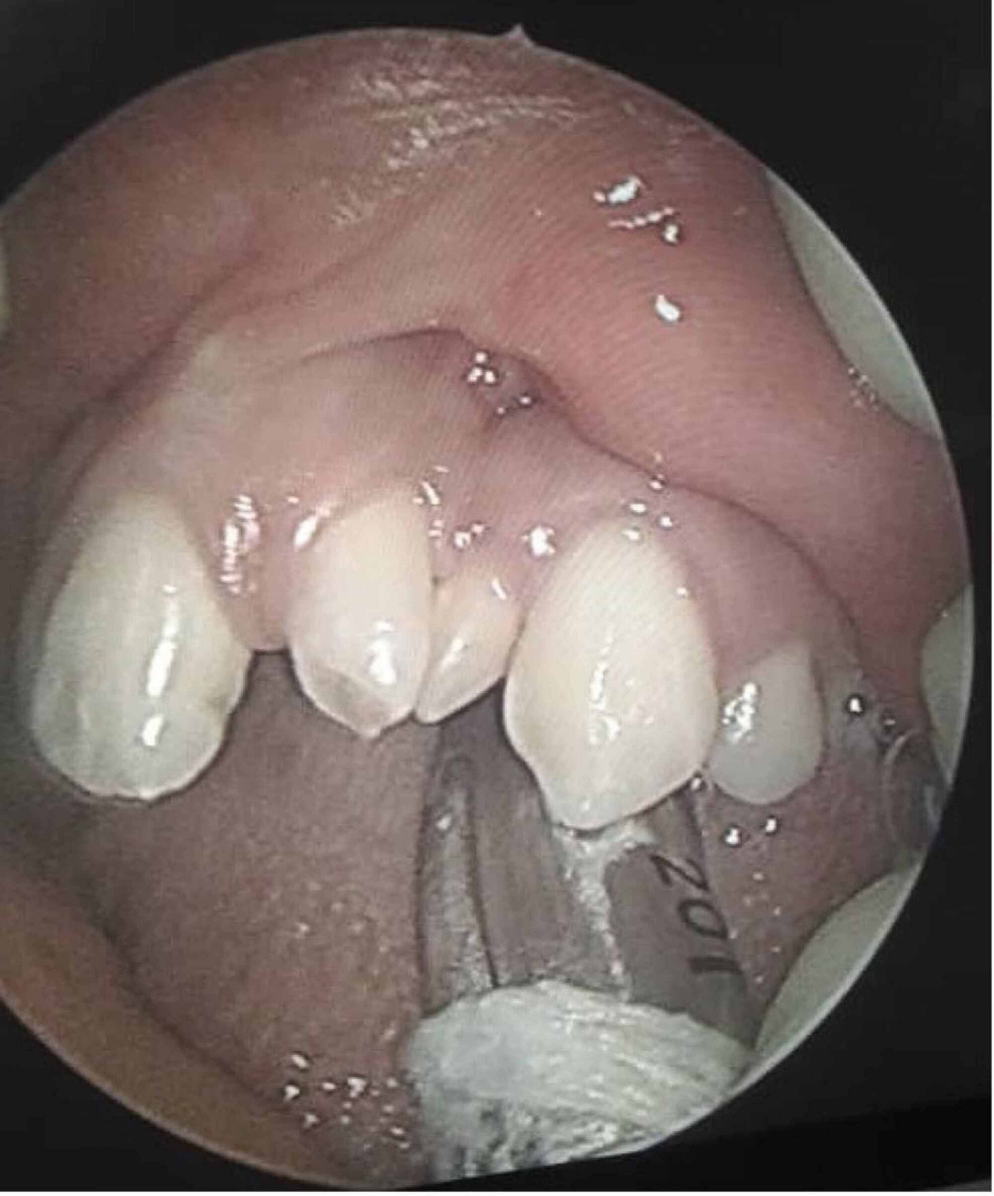
Part of the maxillary arch Canines and the left upper premolar teeth are well formed. There are two malformed incisors between the canines.

She was eventually referred to otolaryngology for further management. Physical examination was unremarkable. She had no cleft lip, and her palate appeared intact. However, there was a slight deformity over her left incisor region, and it appeared to be misaligned. The incisors appeared misaligned (Figure 1). Cold spatula test revealed reduced nasal flow over the left compared to the right. Her facial structures were symmetrical. Cranial nerves were intact. There was no cervical lymphadenopathy.

Rigid nasal endoscopy revealed a rhinolith on the floor of the left nasal cavity with surrounding granulation tissue. The nasal cavities were normal, with clear osteomeatal complexes bilaterally. Nasopharynx was also normal. The lesion was embedded on the floor of the left nasal cavity, and the attempts to remove it were abandoned as the patient was in severe pain.

**Figure 2:**
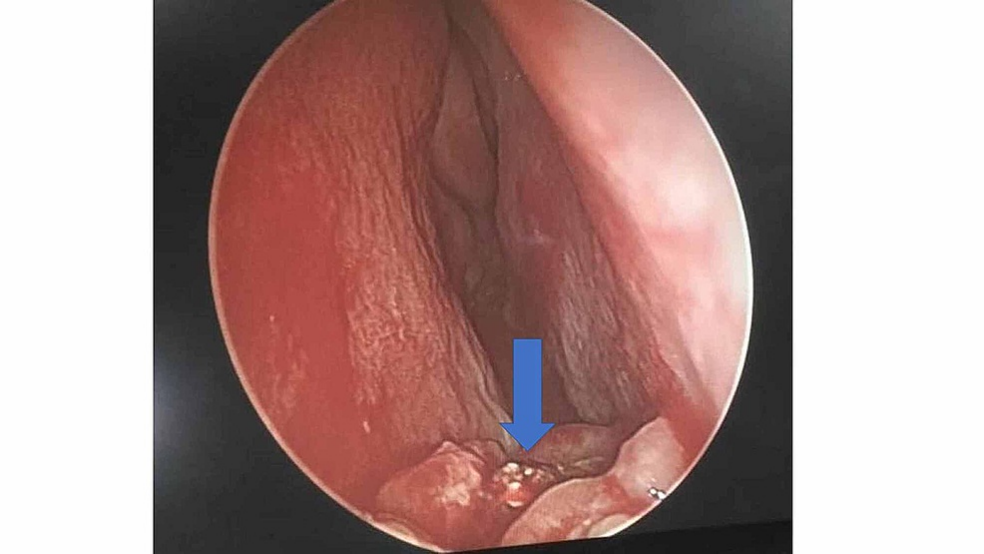
Endoscopic view of the left nasal cavity Part of the granulation tissue was removed, and there was a hard whitish mass (blue arrow) at the floor of the nasal cavity.

**Figure 3:**
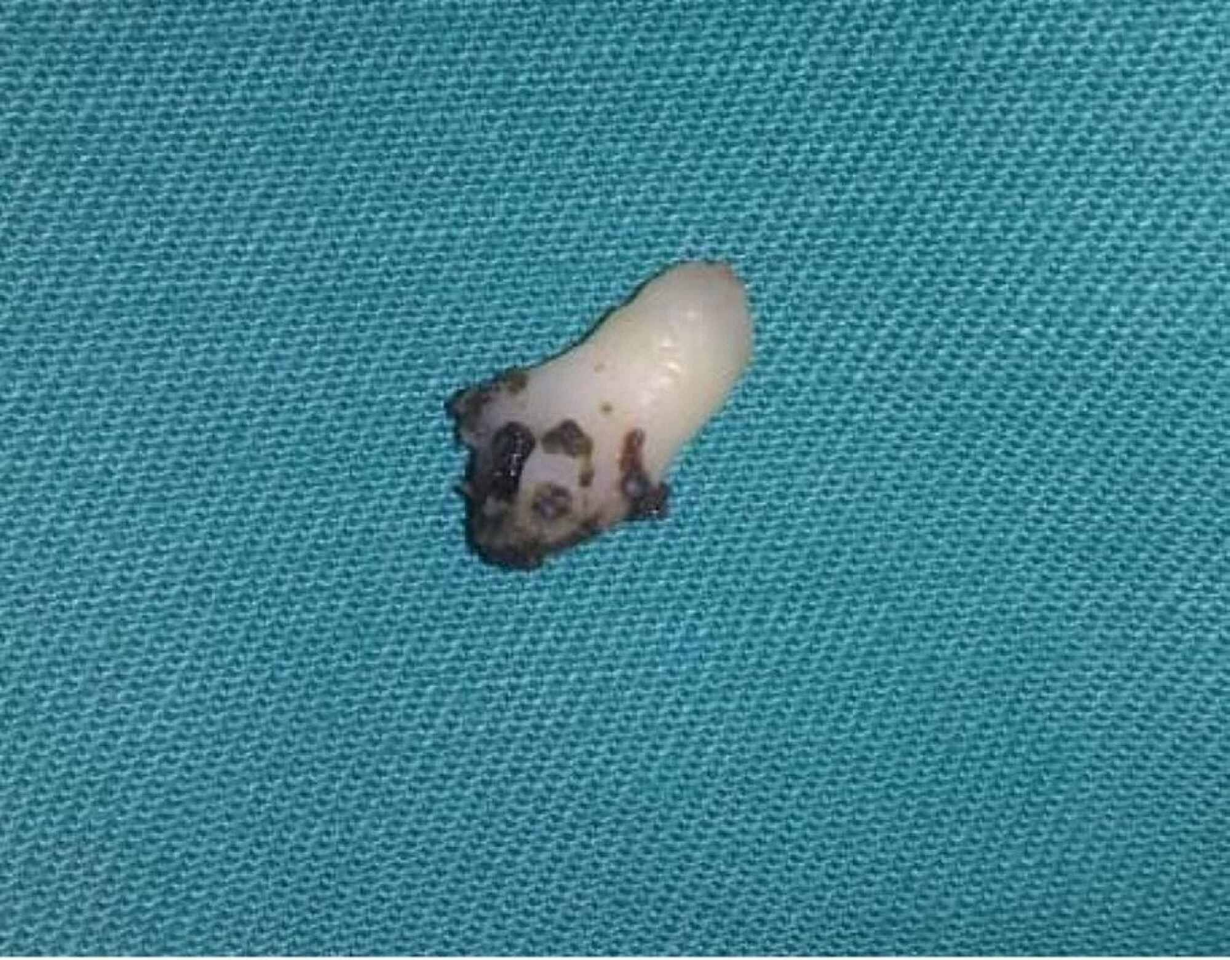
Tooth retrieved from the nasal cavity Tooth revealed typical tooth structures such as the enamel and cementum.

**Figure 4:**
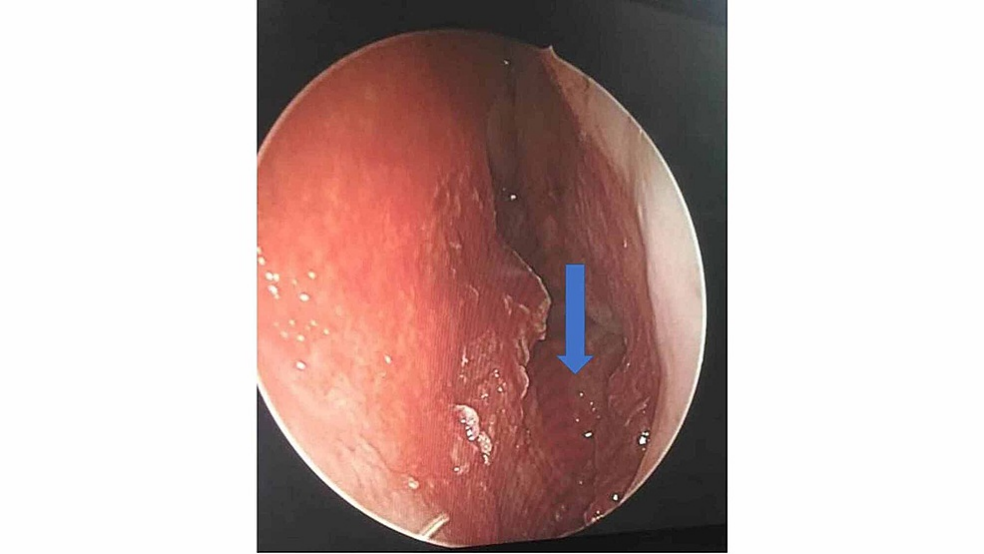
Endoscopic view of the nasal cavity Post removal of the intranasal tooth, the floor of the nasal cavity appeared intact (blue arrow).

She was subjected for examination under general anesthesia whereby the rhinolith was removed under endoscopic guidance. Intraoperatively, the surrounding granulation tissue was removed, and the rhinolith was scooped out with a freer elevator, with minimal manipulation (Figure 2). The rhinolith turned out to be a tooth (Figure 3). The floor of the nasal cavity was intact with no intraoral communication (Figure 4). The histopathology examination of the tissue surrounding the rhinolith was confirmed to be granulation tissue.

Post removal, the patient was well with no more nasal symptoms. She was referred for proper dental assessment; however, due to financial constraints and the resolution of her nasal symptoms, she is yet to present for the follow-up.

## Discussion

A tooth in the nasal cavity is uncommon. Though it occurs more frequently in patients with associated cleft lip or cleft palate [1], the reported incidence is only about 0.1%-1.0% in the general population [2]. Yeung and Lee in 1996 reported 41 well-documented cases [3]. The age of the patients varied from young to old. They were predominantly males [4], and cases were more commonly discovered after adulthood [3].

Most commonly an intranasal tooth occurs unilaterally and as a single tooth. Bilateral involvement and multiple teeth are rare. Lee [5] reported left-side involvement was twice that of the right. An intranasal tooth could be supernumerary, deciduous, or permanent tooth. A supernumerary tooth, which is rudimentary in appearance, is the most common type found intranasally. It is found in the incisor region of the mouth, growing into the floor of the nasal cavity. The exact mechanism is not well defined; it could be idiopathic, associated with developmental disturbances such as cleft lip or cleft palate, crowding of dentition leading to eruption upwards, persistent deciduous teeth [5], or as a consequence of orofacial trauma or osteomyelitis [1].

Patients may be asymptomatic or may present with non-specific nasal symptoms such as unilateral nasal blockage, foul-smelling nasal discharge, epistaxis, facial pain, headache, or even communicating oronasal fistula [1,6,7]. Routine clinical examination or imaging occasionally detects an asymptomatic intranasal tooth [8]. In this case, the patient was asymptomatic for years and only presented with unilateral nasal symptoms for six months, which was misdiagnosed as rhinitis by her local practitioners.

Clinical examination such as anterior rhinoscopy or nasal endoscopy may reveal a hard whitish mass on the floor of the nose, midway in the nasal cavity [5]. However, it may be embedded within the nasal mucosa, or act as a nidus for mineralization, surrounded by granulation tissue, debris, or purulent materials [5]. It is therefore often misdiagnosed, with differential diagnoses including foreign body, rhinolith, odontoma, exostosis, calcifying odontogenic cyst, or even malignant tumor [5,7]. Oropharyngeal examination is important as it may reveal abnormal dentition or palatal defect. Dental and alveolar structures are usually disturbed at the cleft sides, with missing, deformed, or displaced teeth [6].

The diagnosis of rhinolith was made in this case, and no further imaging was done. However, imaging studies such as conventional radiographs or computed tomography could be useful in determining the position of the tooth [4], detecting related complications such as sinusitis, as well as ruling out possible malignancies. They will also be helpful in deciding surgical approaches for removal, be it transnasal or transpalatal excision [7]. Intranasal tooth appears as a dense radio-opacity with similar attenuation as an oral tooth on imaging studies [4]. Dental panoramic radiography, on the other hand, provides detailed information on the dentition of the patient.

In this case, the intranasal tooth removed cannot be determined as supernumerary or malformed permanent tooth due to the absence of proper dental imaging such as orthopantomogram (OPG). OPG provides valuable information regarding other dental anomalies such as hypodontia, impaction, and microdontia. Clinically, her two incisors are missing, but concomitant hypodontia and hyperdontia (simultaneous presence of hypodontia or missing teeth and supernumerary teeth in the same individual) are a very rare dental anomaly with prevalence rate of 0.002%-3.1% [9].

The mainstay of treatment for an intranasal tooth is early removal or extraction to prevent further complications such as abscesses, dental deformities, nasal deformities, oronasal or intranasal fistula, as well as cavernous sinus thrombosis [1,4]. In view of the possibility of underlying chronic inflammation and major complications such as hemorrhage and infection, it is recommended to perform the procedure under general anesthesia [2]. Generally, if the tooth is within the nasal cavity, the removal should be simple [10]. When the tooth has a bony socket at the floor of the nose, it might be difficult to extract. Removal of simple intranasal tooth could be done under direct visualization using nasal speculum or via endoscopic method. The endoscopic method is favorable and the most used approach as it offers good illumination, clear visualization, and precise dissection; thus, limiting injury to the surrounding structures [5]. Levin and Sommer reported all patients had complete resolution of their symptoms following removal of the intranasal tooth [11]. Post removal, infection may resolve with appropriate irrigation and antibiotic treatment.

## Conclusions

Patients with unilateral nasal symptoms not responding to conventional treatment require proper ear, nose, and throat (ENT) evaluation to rule out other pathology. Intranasal tooth is uncommon; however, it should be one of the differential diagnoses to be considered for patients presenting with unilateral nasal symptoms. Early endoscopic evaluation is essential for optimal diagnosis and treatment.
